# Comparison study of the energy and instability of ion-acoustic solitary waves in magnetized electron–positron–ion quantum plasma

**DOI:** 10.1038/s41598-022-23768-8

**Published:** 2022-11-09

**Authors:** W. F. El-Taibany, P. K. Karmakar, A. A. Beshara, M. A. El-Borie, S. A. Gwaily, A. Atteya

**Affiliations:** 1 Department of Physics, Faculty of Science, Damietta University, P.O. Box 34517, New Damietta, Egypt; 2 Department of Physics, Tezpur University, Napaam, Tezpur, 784 028 India; 3 Department of Physics, Faculty of Science, Alexandria University, P.O. 21511, Alexandria, Egypt

**Keywords:** Particle astrophysics, Particle astrophysics

## Abstract

Notably, solitary waves that emerge from the nonlinear properties of plasmas are the main focus of many current studies of localized disturbances in both laboratory and astrophysical plasmas. By applying the reductive perturbation method, we derive the nonlinear homogeneous quantum Zakharov–Kuznetsov (QZK) equation in three-component collisionless quantum plasma consisting of electrons, positrons, and ions in the presence of an external static magnetic field. The solitary wave structures are dependent on the Bohm potential, magnetic field, obliqueness, species Fermi temperatures, and densities. The soliton’s electric field and energy are also derived and investigated, which were found to be reduced as the magnetic field increases. The instability growth rate is also derived by using the small-*k* perturbation expansion method. The previous parameters affect the instability growth rate as well. A comparison of the energy and instability growth rate behaviour against system parameters is carried out. Large energy and large instability growth rate occur at large values of positron density or lower values of ion density. At zero or small rotation angle, both decrease as the magnetic field increases. Our findings could help us understand the dynamics of magnetic white dwarfs, pulsar magnetospheres, semiconductor plasma, and high-intensity laser-solid matter interaction experiments where e-p-i plasma exists.

## Introduction

Studies in the field of multi-component quantum plasmas such as electron-positron-ion (e-p-i) plasmas face more attention and focus lately on the electron-ion quantum plasma due to its prospective application point of view to examine new collective modes and instabilities. Such e-p pair quantum plasma can be present in active galactic cores^1^, discs enclosing black holes^2^, white dwarf environments^3,4^, radiation belts of Van Allen, and intense-laser-plasma fields^5^. The non-linear ion-acoustic (IA) waves phenomena in quantum plasmas, which have positrons, behave differently than the quantum plasma with only electrons and ions. The positron in conventional e-i quantum plasma modulates the characteristics of the linear and non-linear wave modes^6,7^. As well, the e-p plasma has slow and fast time scales depending on whether it is considered as a pair plasma with some concentration of background ions or as an electron-ion plasma with some positron components^8,9^.

In dense degenerate (quantum) e-p-i plasma, despite the quantum effects becoming considerable due to the significant de-Broglie wavelength of the charge carriers (e and p) comparable to the Debye length, i.e., $n_{e0} \uplambda _{B}^{3}\ge 1$, $n_{e0}$ is the equilibrium number density of electrons and $\uplambda _{B}$ is its de-Broglie wavelength, we cannot avoid the ions because of their thermal energy. Therefore, the characteristics of the ions can be understood classically whilst e-p pairs behave as degenerate Fermi gases and can conveniently be described by the Thomas-Fermi model. In addition, the light plasma components are governed by Heisenberg’s uncertainty principle. Following this role, the momentum of greatly confined plasma species tends to be ultra large. This uncurbed momentum of these particles results in generating extreme outward degenerate pressure, which is counter-balanced by the inward gravitational compression. As well, the quantum tunnelling effect can play an important role in exhibiting the collective behavior of the quantum plasma different from the classical one. The utilised quantum hydrodynamic (QHD) model for studying the non-linear propagation of ion-acoustic waves (IAWs) in magnetised e-p-i quantum plasma is extended by adding the quantum statistical pressure term and the quantum diffraction term (the Bohm potential). It’s remarkable that the Bohm potential leads to wave dispersion due to the quantum correlation of density fluctuations associated with the wave nature of the charge carriers.

Over the last two decades, a considerable number of papers have modeled mathematically and numerically the dynamics of e-p-i quantum plasma due to their wide-range applications in different environments. Ghosh and Sahu^10^ utilized the QHD model to characterize a dissipative e-p-i quantum plasma system and obtained the Kadomtsev-Petviashvili-Burger’s equation, which governs the small finite amplitude of 2D ion-acoustic solitary and shock waves. Haque^11^ investigated the impact of the exchange-correlation and pressure degeneracy on the behavior of the IAWs in an inhomogeneous dense e-p-i plasma. Saha et al.^12^ presented the bifurcation analysis of quantum ion-acoustic kink, anti-kink, and periodic waves in a dense degenerate plasma including positive ions, electrons, and positrons. Moreover, Iqbal et al.^13^ considered electron and positron as two different fluids in their study, which deals with magnetosonic waves in spin-polarized e-p-i quantum plasma. This study demonstrated that the polarization due to spin and positron concentration minimizes the phase velocities of the obliquely propagating spectra, as well as the obliqueness effect, which reinforces the frequency of all modes. Saha and Banerjee introduced the background of dynamical systems, waves, and oscillations in plasmas ^14^. Basic concepts of dynamical systems and phase plane analysis for the study of dynamical properties of nonlinear waves in plasmas were presented. 

To characterize the mode of change of the behavior of the IAWs structures in e-p-i quantum plasmas subject to a magnetic field, the nonlinear Zakharov–Kuznetsov (ZK) equation is derived via applying the reductive perturbation scheme. The ZK equation, which is a well-studied canonical three-dimensional extension of the Korteweg-de Vries equation, identifies the extent of the weak non-linearity and dispersion of such wave modes. The ZK equation for IAWs was derived for magnetized plasma whose constituents are cold ions and kappa-distributed electrons^15^. As a first step, the bifurcation theory was used to test the existence of solitary wave and periodic traveling wave solutions. The propagation characteristics of the solitary structure in the presence of the Landau quantization in dense e-p-i plasmas through the ZK equation are investigated by Hussain et al.^16^. On the other hand, Soltani et al.^17^ derived the ZK equation for the oblique propagation of the electrostatic solitary waves in a magnetized quantum plasma consisting of dynamic relativistic degenerate electrons and positrons and weakly relativistic ions, and hence, slow and fast acoustic modes are found to be present in this quantum plasma model. As well, Mohsenpour et al.^18^ considered negative ion plasma in their survey of ion-acoustic quantum solitary modes in a polarised quantum plasma comprising relativistic degenerate electrons and positrons governed by the ZK equation.

An essential field of plasma physics is the instability of plasma systems. Plasma instability is a region where turbulence occurs due to fluctuations in the characteristics of a dense plasma (e.g. temperature, density, electric field, magnetic field). It usually only makes sense to analyze the instability of a plasma once it has been established that the plasma is in an equilibrium state. So, instability analysis defines whether a small perturbation will grow, oscillate, or be damped out. Subsequently, plasma instabilities can be divided into two general groups: hydrodynamic and kinetic instabilities, which in turn are categorized into other different modes. The small-k expansion technique is one of a variety of methods used to deduce the growth rate of the unstable non-linear wave structures of the plasma model. The streaming instability analysis is carried out by Iqbal et al.^19^ for transverse waves of counter-streaming e-p pairs parallel to the magnetic field and ions in a magneto-quantum plasma. They presented a new positron streaming-driven transverse mode. Under the influence of the chemical potential of electrons, the instability gauge and its growth rate of the ZK equation for isothermal IA solitons in a magnetized ultra-relativistic degenerate multicomponent quantum plasma were investigated by El-Shamy et al.^20^. Thereafter, Khanum et al.^21^ studied the spin-polarized e-p-i plasma and examined the spin effect on the counter-streaming instability, as well as discussed the impact of positron number density, streaming speed, and polarization due to spin on the frequency of waves and the growth rate. Behery and Zaghloul^22^ used Chandrasekhar’s equation of state for the degenerate relativistic electrons and positrons while ions are presented as fixed and uniform in space, to get a non-linear ZK equation of electrostatic waves in magnetized quantum plasma. The small-*k* expansion method is applied to examine the instability criteria of such oblique waves.

A few authors are concerned with instability analysis and determining the growth rate for solitary wave solutions for the four-dimensional ZK equation in e-p-i quantum plasma. In this paper, we will analyse the spectral instability of the independent solutions of the ZK equation for long-wavelength transverse perturbations in e-p-i quantum magnetised plasma by following the procedure provided by Hongsit et al.^23^, which depends on the small-*k* expansion technique.

 The layout of this paper can be mentioned as follows: Section two presents the magneto-quantum hydrodynamic equations that model our system. Section three sustains forward to derive the nonlinear (3+1) ZK equation. In section four, we obtained a stationary plane solitary solution of the quantum-ZK (QZK) equation. We then carry out our transverse instability analysis for the QZK equation in section five. Section six is limited to numerical investigation and discussion. What is left for conclusions has been mentioned in section seven.

## Basic equations of the model

We consider the nonlinear propagation of quantum ion-acoustic solitary waves (QIASWs) in a three-component collisionless quantum plasma consisting of electrons, positrons, and ions in the presence of an external static magnetic field of flux density ${\mathbf {B}}_{{\mathbf {o}}}=B_{o}{\hat{z}}$, where ${\hat{z}}$ is the unit vector along the z-direction. Electrons and positrons are considered inertialess by supposing that the phase velocity of the traveling wave is much less than the Fermi velocities of electrons and positrons, respectively. The hydrodynamics of QIASW’s in e-p-i plasma is governed by a set of equations as follows^24^,

1 $$\begin{aligned} \left. \begin{array}{c} \frac{\partial n_{i}}{\partial t}+\nabla \cdot \left( n_{i}{\mathbf {u}} _{i}\right) =0,\\ \frac{\partial {\mathbf {u}}_{i}}{\partial t}+\left( {\mathbf {u}}_{i}\cdot \nabla \right) {\mathbf {u}}_{i}=-\nabla \phi +\omega \left( {\mathbf {u}}_{i} \times {\hat{z}}\right) ,\\ \nabla ^{2}\phi =\left( 1+p\right) n_{e}-pn_{p}-n_{i},\\ 2\phi =n_{e}^{2}-1-H_{e}^{2}\left( \frac{1}{\sqrt{n_{e}}}\nabla ^{2}\sqrt{n_{e}}\right) ,\\ 2\phi =\sigma -\sigma n_{p}^{2}+H_{p}^{2}\left( \frac{1}{\sqrt{n_{p}}} \nabla ^{2}\sqrt{n_{p}}\right) , \end{array} \right\} \end{aligned}$$

where $n_{\alpha }$ $\left( \alpha =\text {e for electrons, i for ions, and p for positrons}\right)$ is the number density normalized by their unperturbed number density $n_{\alpha 0}$, ${\mathbf {u}}_{i}$ is the ion fluid velocity normalized by the ion acoustic Fermi speed $c_{i}=\sqrt{2K_{B}T_{Fe}\diagup m_{i}}$; $\phi$ is the electrostatic wave potential normalized by $\left( 2K_{B}T_{Fe}\diagup e\right)$; *e* is the elementary charge; $\omega$ is the ion cycltron frequency $\left( \omega =eB_{0}\diagup m_{i}\right)$ normalized by the ion plasma frequency $\omega _{pi}=\sqrt{4\pi n_{i0} e^{2}\diagup m_{i}}$. The time and space coordinates are in units of the ion gyroperiod $\omega _{pi}^{-1}=\sqrt{m_{i}\diagup 4\pi n_{i0}e^{2}}$, and the ion gyroradius $\uplambda _{i}=\sqrt{2K_{B}T_{Fe}\diagup 4\pi n_{i0}e^{2}}$, respectively. As well, $\sigma$ is a numerical factor expresses the ratio of electron to positron Fermi temperature $\left( T_{Fe}\diagup T_{Fp}\right)$ and $p=n_{p0}\diagup n_{i0}$. The dimensionless quantum parameter $H_{e,p}$ is given by $H_{e,p}=\left( h\omega _{pi}\diagup 2\pi c_{i}^{2}\sqrt{m_{i}m_{e,p} }\right)$, where *h* is Planck’s constant and $m_{e,p,i}$ is the mass of electron, positron and ion, respectively. Furthermore, the quantum diffraction term in equation (1) has been neglected as $m_{e,p}\ll m_{i}$ and we do not over look the mention of the equilibrium requirement which states that $n_{e0}=n_{i0}+n_{p0}$.

## Deduction of non-linear (3+1) quantum ZK equation

      We follow the reductive perturbation theory to the system of equations to investigate the non-linear spread of QIASWs in (*x*, *y*, *z*, and *t*) dimensions with a small finite amplitude. To achieve that, we primarily expand the dependent plasma variables about their equilibrium point as a power series of $\epsilon$ as follows^25^

2 $$\begin{aligned} \left. \begin{array}{c} n_{e,p,i}=1+\epsilon n_{e,p,i1}+\epsilon ^{2}n_{e,p,i2}+...,\\ u_{iz}=\epsilon u_{iz1}+\epsilon ^{2}u_{iz2}+...,\\ u_{ix,y}=\epsilon ^{\frac{3}{2}}u_{ix,y1}+\epsilon ^{\frac{4}{2}}u_{ix,y2} +...,\\ \phi =\epsilon \phi _{1}+\epsilon ^{2}\phi _{2}+..., \end{array} \right\} \end{aligned}$$

where $\epsilon$ is a dispersion parameter from equilibrium, it measures the perturbation amplitude $\left( 0<\epsilon \le 1\right)$. Thus, we stretch the independent dimensional variables to a moving frame of wave phase speed $\uplambda$ which is normalized by the ion-acoustic speed $\left( c_{i}\right)$ as,

3 $$\begin{aligned} X=\epsilon ^{\frac{1}{2}}x Y=\epsilon ^{\frac{1}{2}}y Z=\epsilon ^{\frac{1}{2}}\left( z-\uplambda t\right) ,\tau =\epsilon ^{\frac{3}{2}}t. \end{aligned}$$

By substituting Eqs. (2) and (3) into the system equations, Eq. (1), we get a set of equations in ordered series of powers of $\epsilon$. Hence, collecting the coefficients of the lowest order of $\epsilon$ for all the species, we get the following relations for the lowest order perturbed quantities

4 $$\begin{aligned} n_{i1}=\frac{1}{\uplambda },u_{iZ1}=\frac{1}{\uplambda ^{2} }\phi _{1},n_{e1}=\phi _{1},n_{p1}=-\frac{1}{\sigma }\phi _{1}. \end{aligned}$$

On substitution in the Poisson’s equation, we get the neutrality condition in the form

5 $$\begin{aligned} \left( 1+p\right) n_{e1}=pn_{p1}+n_{i1}, \end{aligned}$$

from which the phase speed of the perturbation wave could be evaluated via the following dispersion relation,

6 $$\begin{aligned} \uplambda =\sqrt{\frac{\sigma }{\sigma +p\left( 1+\sigma \right) }.} \end{aligned}$$

Also, by collecting the coefficients of the next higher order of $\epsilon$, we obtain the following relations

7 $$\begin{aligned} u_{iX1}=-\frac{1}{\omega }\frac{\partial \phi _{1}}{\partial Y},u_{iY1}=\frac{1}{\omega }\frac{\partial \phi _{1} }{\partial X},u_{iZ1}=\frac{1}{\uplambda }\phi _{1}. \end{aligned}$$

The coefficients of the next higher orders of $\epsilon$ lead to equations that are solved to obtain the second-order perturbed quantities. This quantity is collected in the $\epsilon ^{2}$ order of the Poisson’s equation represented as

8 $$\begin{aligned} \nabla ^{2}\phi _{1}=\left( 1+p\right) n_{e2}-pn_{p2}-n_{i2}. \end{aligned}$$

Taking into account the same order gives the relations

9 $$\begin{aligned} \left. \begin{array}{c} \phi _{2}+\sigma n_{p2}=-\frac{\sigma }{2}n_{p1}^{2}+\frac{H_{p}^{2}}{4} \nabla ^{2}n_{p1,}\\ \phi _{2}-n_{e2}=-\frac{1}{2}n_{e1}^{2}+\frac{H_{e}^{2}}{4}\nabla ^{2}n_{e1}. \end{array} \right\} \end{aligned}$$

Thus, The series of partial derivative equations can be algebraically manipulated to realize the QZK equation as,

10 $$\begin{aligned} \frac{\partial \phi _{1}}{\partial \tau }+A\phi _{1}\frac{\partial \phi _{1} }{\partial Z}+B\frac{\partial ^{3}\phi _{1}}{\partial Z^{3}}+C\frac{\partial }{\partial Z}\left( \frac{\partial ^{2}}{\partial X^{2}}+\frac{\partial ^{2} }{\partial Y^{2}}\right) \phi _{1}=0, \end{aligned}$$

with nonlinear, longitudinal dispersion, and the transverse coefficients *A*, *B*, and *C*, respectively, which are given by,

11 $$\begin{aligned} \left. \begin{array}{c} A=\frac{\uplambda ^{3}}{2}\left( \frac{3}{\uplambda ^{4}}+p(\frac{\sigma ^{2} -1}{\sigma ^{2}})+1\right) ,\\ B=\frac{\uplambda ^{3}}{2}\left( 1-\left( 1+p\right) \frac{H_{e}^{2}}{4} -\frac{pH_{p}^{2}}{4\sigma ^{2}}\right) ,\\ C=B+\frac{\uplambda ^{5}}{2\omega ^{2}}\left( p\left( \frac{\sigma +1}{\sigma }\right) +1\right) . \end{array} \right\} \end{aligned}$$

## Solitary wave analysis

The solitary wave solution of Eq. (10) arises from the balance between nonlinearity and dispersive effects, we shall first follow the transformation of the independent variables^26–28^ through rotating the coordinate axes (*X*, *Z*) by an angle $\theta$ and renaming *Y* and *T*, the coordinate transformations are defined as

12 $$\begin{aligned} \left. \begin{array}{c} \zeta =X\cos \theta -Z\sin \theta ,\\ \xi =X\sin \theta +Z\cos \theta ,\\ \eta =Y,\text {and }\tau =T. \end{array} \right\} \end{aligned}$$

Applying these transformations to the QZK Eq. (10), we obtain

13 $$\begin{aligned} \left. \begin{array}{c} \frac{\partial \phi ^{(1)}}{\partial \tau }+S_{1}\phi ^{(1)}\frac{\partial \phi ^{(1)}}{\partial \xi }+S_{2}\frac{\partial ^{3}\phi ^{(1)}}{\partial \xi ^{3} }+S_{3}\phi ^{(1)}\frac{\partial \phi ^{(1)}}{\partial \zeta }+S_{4}\frac{\partial ^{3}\phi ^{(1)}}{\partial \zeta ^{3}}\\ +S_{5}\frac{\partial ^{3}\phi ^{(1)}}{\partial \xi ^{2}\partial \zeta }+S_{6} \frac{\partial ^{3}\phi ^{(1)}}{\partial \xi \partial \zeta ^{2}}+S_{7} \frac{\partial ^{3}\phi ^{(1)}}{\partial \xi \partial \eta ^{2}}+S_{8}\frac{\partial ^{3}\phi ^{(1)}}{\partial \zeta \partial \eta ^{2}}=0, \end{array} \right\} \end{aligned}$$

where

14 $$\begin{aligned} \left. \begin{array}{c} S_{1}=A\cos \theta ,S_{2}=B\cos ^{3}\theta +C\sin ^{2}\theta \cos \theta ,\\ S_{3}=-A\sin \theta ,S_{4}=-B\sin ^{3}\theta -C\cos ^{2}\theta \sin \theta ,\\ S_{5}=2C(\sin \theta \cos ^{2}\theta -\frac{1}{2}\sin ^{3}\theta )-3B\cos ^{2} \theta \sin \theta ,\\ S_{6}=-2C(\sin ^{2}\theta \cos \theta -\frac{1}{2}\cos ^{3}\theta )+3B\sin ^{2} \theta \cos \theta ,\\ S_{7}=C\cos \theta ,S_{8}=-C\sin \theta . \end{array} \right\} \end{aligned}$$

The steady-state solution of QZK equation can be taken as

$$\begin{aligned} \phi ^{(1)}=\phi _{0}(\rho ), \end{aligned}$$

where $\rho =\xi -M\tau$, and *M* is the Mach number normalized by the ion-acoustic Fermi speed $c_{i}$. So, Eq. (13) can be written as^29^

15 $$\begin{aligned} -M\frac{d\phi _{0}}{d\rho }+S_{1}\phi _{0}\frac{d\phi _{0}}{d\rho }+S_{2} \frac{d^{3}\phi _{0}}{d\rho ^{3}}=0. \end{aligned}$$

Integrating and applying appropriate boundary conditions, we obtain the QIASWs pulse solution as

16 $$\begin{aligned} \phi _{0}(\rho )=\phi _{m}{\text {sech}}^{2}(\frac{\rho }{W}), \end{aligned}$$

where $\varphi _{m}$ and *W* are the amplitude and the width of the solitary wave, respectively; these are expressed as

17 $$\begin{aligned} \phi _{m}=3M/S_{1}\text { and }W=2\sqrt{S_{2}/M}. \end{aligned}$$

The associated electric field could be deduced and take the form

18 $$\begin{aligned} E_{0}(\rho )=-\bigtriangledown \phi _{0}(\rho )=\frac{2\phi _{m}}{W} {\text {sech}}^{2}(\frac{\rho }{W})\tanh (\frac{\rho }{W}). \end{aligned}$$

It is clear from Eqs. (14) and (17) that both the amplitude and the width of the solitary wave depend on the electron and positron Bohm potential and Fermi temperatures. Also, the positron and ions equilibrium densities have considerable effects. In addition to the above, the energy is also an important feature, which can be calculated as reported by Ko and Kuehl^25,30^ as

19 $$\begin{aligned} E_{n}&= {\displaystyle \int \limits _{-\infty }^{\infty }} \frac{\phi _{0}^{2}(\rho )}{\uplambda ^{2}}d\rho ,\nonumber \\ E_{n}&=\frac{4W\phi _{m}^{2}}{3\uplambda ^{2}}. \end{aligned}$$

It reveals to what extent the confined charged particles acquire energy from the wave.

## Instability analysis

In the stability investigation of this solution, we employ the small-*k* expansion perturbation method. We assume that^28,31^

20 $$\begin{aligned} \phi ^{\left( 1\right) }=\phi _{0}(\rho )+\Phi (\rho ,\zeta ,\eta ,\tau ), \end{aligned}$$

where the oblique plane propagating long-wavelength-wave takes the symbol $\Phi$ that can be represented as

21 $$\begin{aligned} \Phi (\rho ,\zeta ,\eta ,\tau )=\psi (\rho )\exp i[k(l_{\xi }\rho +l_{\zeta } \zeta +l_{\eta }\eta )-\gamma \tau ], \end{aligned}$$

in which $l_{\xi }^{2}+l_{\zeta }^{2}+l_{\eta }^{2}=1$, $\psi (\rho )$, and $\gamma$ can be expanded by assuming small values of *k* to the form

22 $$\begin{aligned} \left. \begin{array}{c} \psi (\rho )=\psi _{o}+k\psi _{1}+k^{2}\psi _{2}+...,\\ \gamma =k\gamma _{1}+k^{2}\gamma _{2}+.... \end{array} \right\} \end{aligned}$$

Substituting Eq. (20) into (13) to get the linearized QZK equation as

23 $$\begin{aligned} \left. \begin{array}{c} \frac{\partial \Phi }{\partial \tau }-M\frac{\partial \Phi }{\partial \rho }+S_{1} \phi _{0}\frac{\partial \Phi }{\partial \rho }+S_{2}\frac{\partial ^{3}\Phi }{\partial \rho ^{3}}\\ +S_{3}\phi _{0}\frac{\partial \Phi }{\partial \zeta }+S_{4}\frac{\partial ^{3}\Phi }{\partial \zeta ^{3}}+S_{5}\frac{\partial ^{3}\Phi }{\partial \rho ^{2} \partial \zeta }+S_{6}\frac{\partial ^{3}\Phi }{\partial \rho \partial \zeta ^{2} }+S_{7}\frac{\partial ^{3}\Phi }{\partial \rho \partial \eta ^{2}}+S_{8} \frac{\partial ^{3}\Phi }{\partial \zeta \partial \eta ^{2}}=0. \end{array} \right\} \end{aligned}$$

Substituting Eqs. (21 and 22) into (23) and equating the same-order of *k* power coefficients leads to

24 $$\begin{aligned} (-M+S_{1}\phi _{0})\psi _{o}+S_{2}\frac{d^{2}\psi _{o}}{d\rho ^{2}}=C^{^{\prime }}, \end{aligned}$$

where $C^{^{\prime }}$ is the integration constant. The homogeneous part of Eq. (24) has two independent linear solutions which can be expressed as^28^,

25 $$\begin{aligned} f=\frac{d\psi _{0}}{d\rho },g=f\int ^{\rho }\frac{d\rho }{f^{2}}. \end{aligned}$$

Consequently, the general solution can be arises to take the next form

26 $$\begin{aligned} \psi _{0}=C_{1}f+C_{2}g-C^{^{\prime }}f\int ^{\rho }\frac{g}{S_{2}}d\rho +C^{^{\prime }}g\int ^{\rho }\frac{f}{S_{2}}d\rho , \end{aligned}$$

where $C_{1}$ and $C_{2}$ are the constants of the integration and ${\hat{W}}$ is the Wronskian that can be defined as

$$\begin{aligned} {\hat{W}}=f(dg/d\rho )-g(df/d\rho ). \end{aligned}$$

The zeroth-order general solution equation can be finally simplified to the following equation

27 $$\begin{aligned} \psi _{0}=C_{1}f. \end{aligned}$$

The first and second-order equations from Eqs. (21–23) can be obtained, where the dispersion relation can be represented from their solutions as follow

28 $$\begin{aligned} \gamma _{1}=\Delta -Ml_{\xi }+\sqrt{\Delta ^{2}-\Gamma }, \end{aligned}$$

where

29 $$\begin{aligned} \left. \begin{array}{c} \Delta =\frac{2}{3}(\mu _{1}\phi _{m}-2\mu _{2}/W^{2}),\\ \Gamma =\frac{16}{45}(\mu _{1}^{2}\phi _{m}^{2}-3\mu _{1}\mu _{2}\phi _{m} /W^{2}-3\mu _{2}^{2}/W^{4}+12S_{2}\mu _{3}/W^{4}),\\ \mu _{1}=(S_{1}l_{\xi }+S_{3}l_{\zeta })\text {, }\mu _{2}=(3S_{2}l_{\xi } +S_{5}l_{\zeta }),\\ \text {and }\mu _{3}=(3S_{2}l_{\xi }^{2}+2S_{5}l_{\xi }l_{\zeta }+S_{6}l_{\zeta }^{2}+S_{7}l_{\eta }^{2}). \end{array} \right\} \end{aligned}$$

Hence, from Eq. (28), we notice that instability occurs if the condition $\Gamma -\Delta ^{2}>0$ is satisfied. We obtain the instability growth rate, *Gr*, to be represented as

30 $$\begin{aligned} Gr=\sqrt{\Gamma -\Delta ^{2}}. \end{aligned}$$

This instability growth rate depends on the system parameters that will be discussed in the next section.

## Numerical investigations and discussion

Using the reductive perturbation method leads to deriving the dispersion relation in the case of linear analysis, while the nonlinear analysis gives the 3+1 dimension QZK equation in a three-component collisionless magnetized quantum e-p-i plasma. The solution of this evolved equation gives a solitary wave and its associated electric field and energy were also derived. The small-*k* expansion technique was employed to examine the multi-dimensional instability of the QIASWs that are controlled by the previous QZK equation^32,33^. The results of this investigation may be summarized as follows:

The QIASWs properties such as speed, polarity, amplitude, and width are found to be slightly affected by the electron and positron Fermi temperatures, positron and ion equilibrium densities. Also, the electron and positron Bohm potentials affect the formed QIASWs. Consequently, these parameters affect the associated electric field and wave energy.

**Figure 1:**
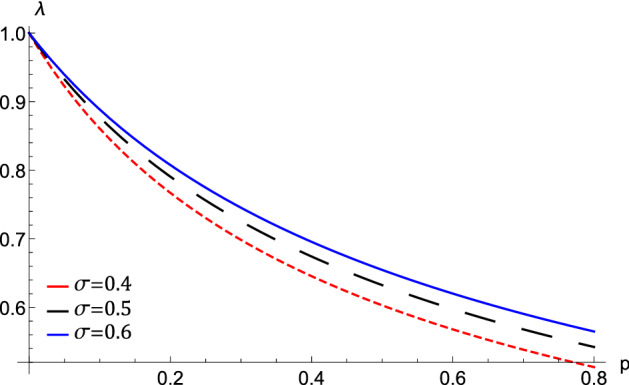
The phase velocity $\uplambda$, variation against *p* at different values of $\sigma$.

The effect of varying the value of the positron to ion density ratio via *p* on the phase speed, $\uplambda$ for different values of the electron to positron Fermi temperature ratio, $\sigma$, is depicted in Fig. 1. We notice that the phase speed enhances with the increase of $\sigma$, while it shrinks with the increase of *p*. i.e., the QIASWs will be faster if the electron Fermi temperature and the ion density become higher. The same effect is obtained when the positron density decreases. This is physical due to the effect of electron pressure on the restoring force, which is enhanced by increasing the electron Fermi temperature. While the inertia is enhanced by increasing the ion density.

**Figure 2:**
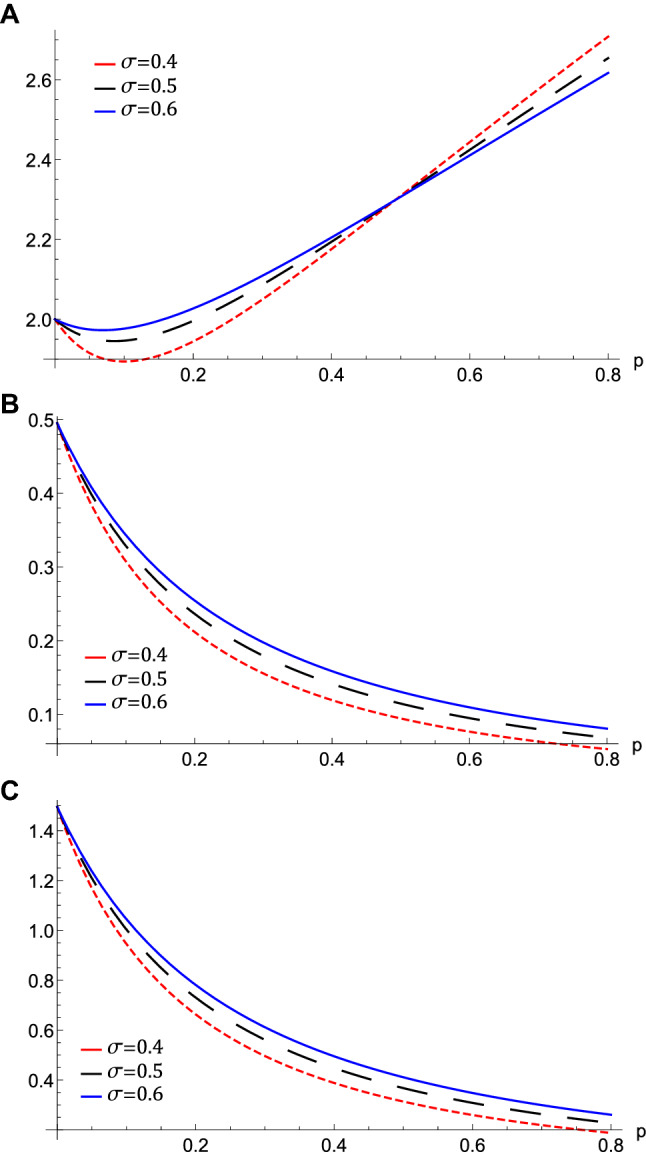
(**A**) The variation of the nonlinear term *A*, (**B**) The longitudinal dispersive term *B*, (**C**) The transverse dispersive term *C* represented by equation $\left( 11\right)$ against *p* for different values of $\sigma$ at $\omega =0.5$, $\theta =20$, $H_{e}=0.2$, and $H_{p}=0.4$.

**Figure 3:**
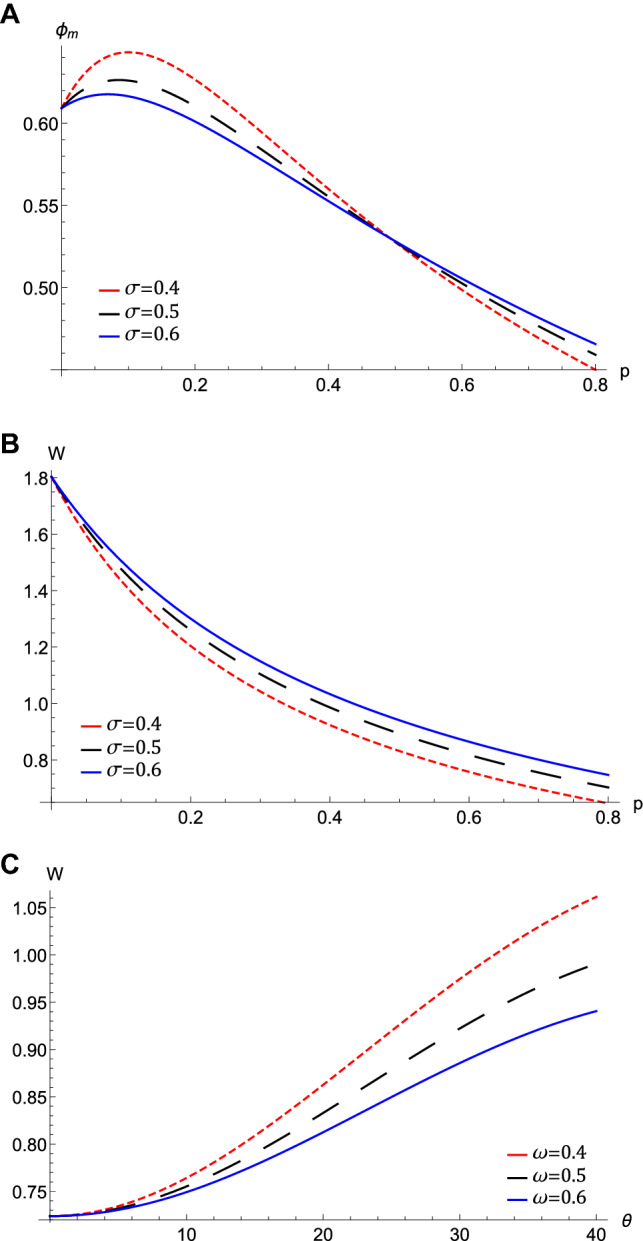
The variation of the QIASWs (**A**) the amplitude $\phi _{m}$ against *p* for different values of $\sigma$ at $\omega =0.5$, $\theta =20$, (**B**) the width *W* against *p* for different values of $\sigma$ at $\omega =0.5$, $\theta =20$, (**C**) the width *W* against $\theta$ for different values of $\omega$ at $\sigma =0.5$, $p=0.7$ with all at $H_{e}=0.4$, and $H_{p}=0.6$.

**Figure 4:**
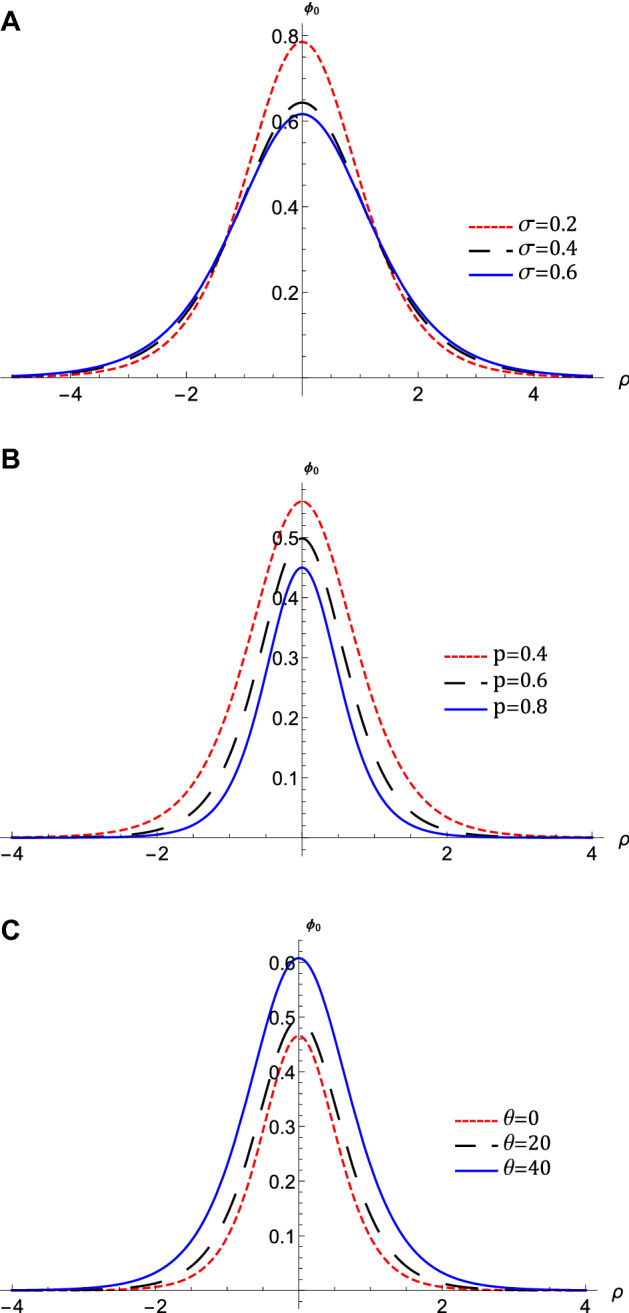

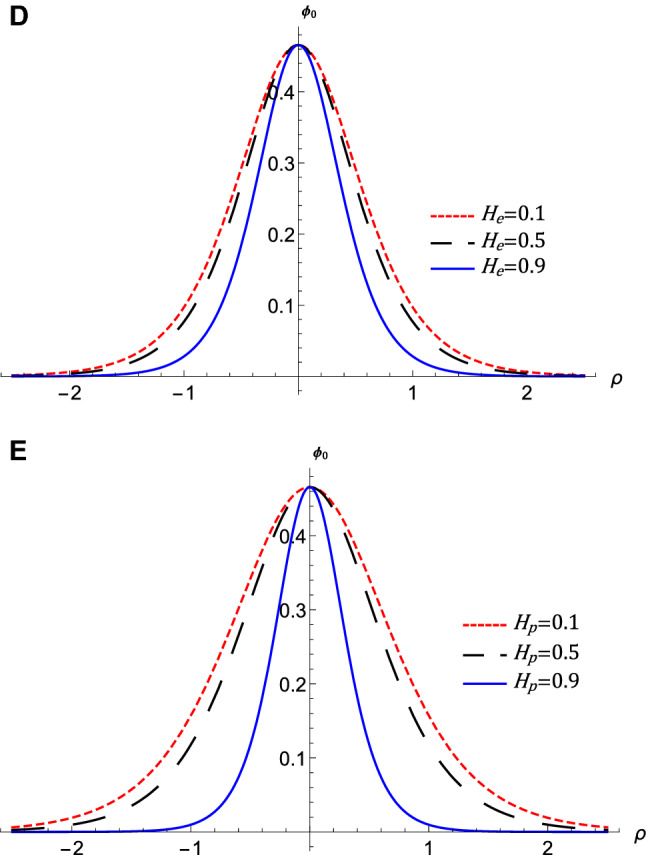
The variation of the QIASWs potential $\phi _{0}$ that represented by equation $\left( 16\right)$ against $\rho$ at $\omega =0.5$, *M* $=0.4$, (**A**) for different values of $\sigma$ with $p=0.1$, $\theta$ $=1$, $H_{e}=0.1$, and $H_{p}=0.6$, (**B**) for different values of *p* with $\sigma =0.4$, $\theta$ $=1$, $H_{e}=0.1$, and $H_{p}=0.6$, (**C**) for different values of $\theta$ with $p=0.7$, $\sigma$ $=0.4$, $H_{e}=0.1$, and $H_{p}=0.6$, (**D**) for different values of $H_{e}$ with $p=0.7$, $\sigma =0.4$, $\theta$ $=1$, and $H_{p}=0.6$, (**E**) for different values of $H_{p}$ with $p=0.7$, $\sigma =0.4$, $\theta$ $=1$, and $H_{e}=0.6$.

**Figure 5:**
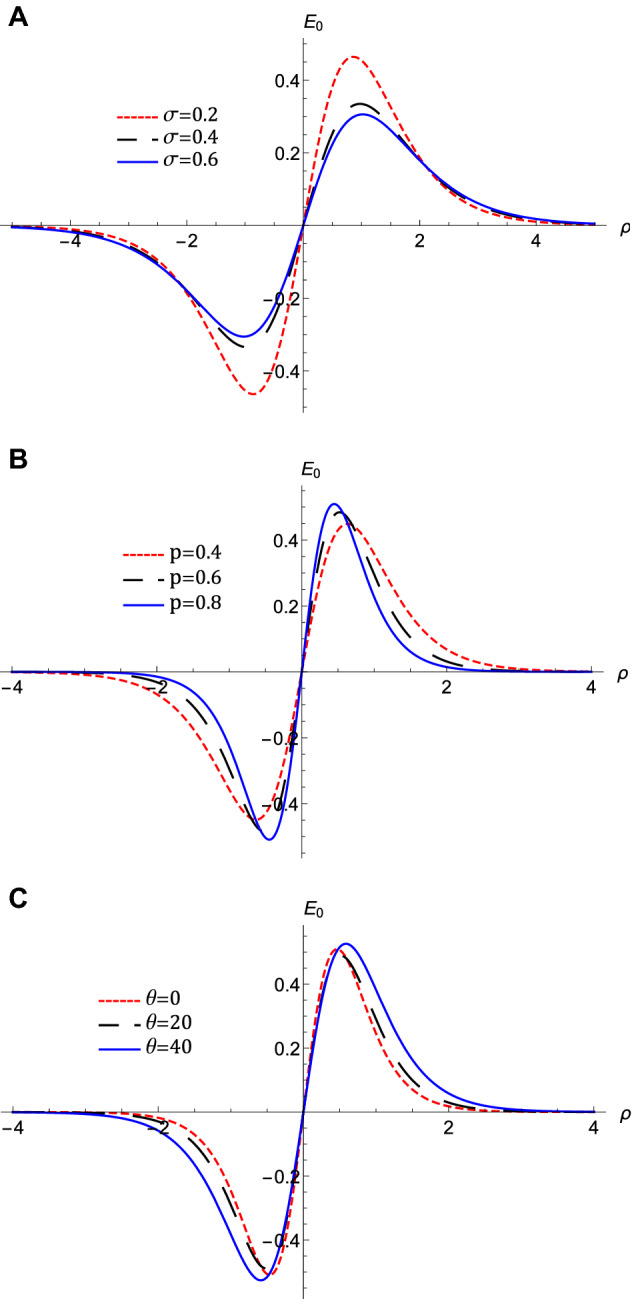

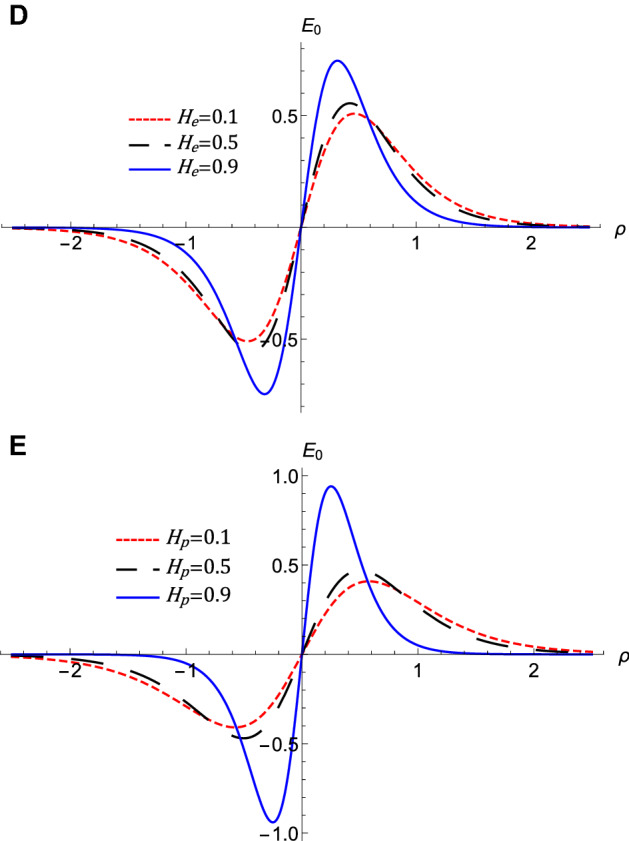
The evolution of the associated electric field, $E_{0}$ of QIASWs that represented by equation $\left( 18\right)$ with $\rho$ for the potentials those represented by Fig. 5, (**A**) for different values of $\sigma$ with $p=0.1$, $\theta$ $=1$, $H_{e}=0.1$, and $H_{p}=0.6$, (**B**) for different values of *p* with $\sigma =0.4$, $\theta$ $=1$, $H_{e}=0.1$, and $H_{p}=0.6$, (**C**) for different values of $\theta$ with $p=0.7$, $\sigma$ $=0.4$, $H_{e}=0.1$, and $H_{p}=0.6$, (**D**) for different values of $H_{e}$ with $p=0.7$, $\sigma =0.4$, $\theta$ $=1$, and $H_{p}=0.6$, (**E**) for different values of $H_{p}$ with $p=0.7$, $\sigma =0.4$, $\theta$ $=1$, and $H_{e}=0.6$.

**Figure 6:**
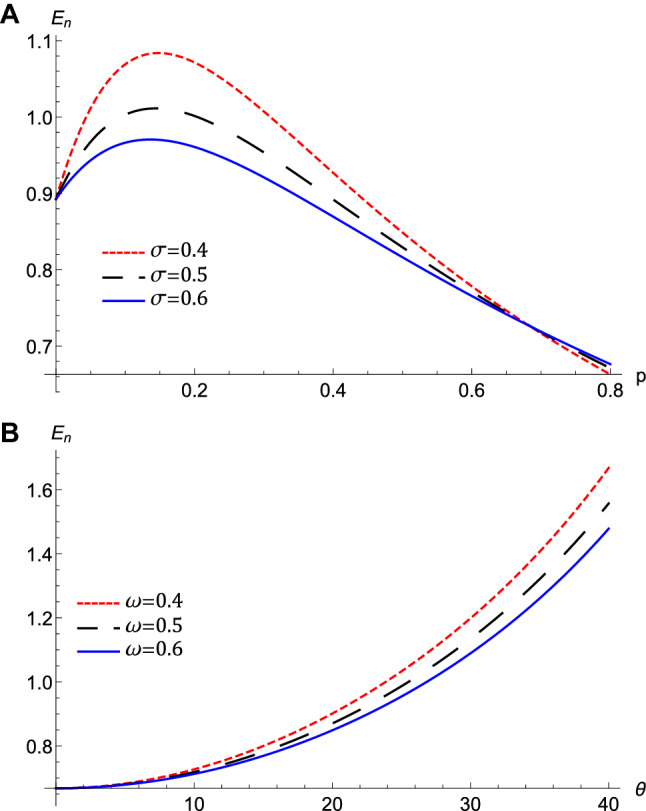
The evolution of the energy $E_{n}$ of the QIASWs that represented by equation $\left( 19\right)$ at *M* $=0.4$, $H_{e}=0.4$, and $H_{p}=0.6$, (**A**) against *p* for different values of $\sigma$ at $\omega =0.5$, $\theta =20$, (**B**) against $\theta$ for different values of $\omega$ at $\sigma =0.5$, $p=0.7$.

**Figure 7:**
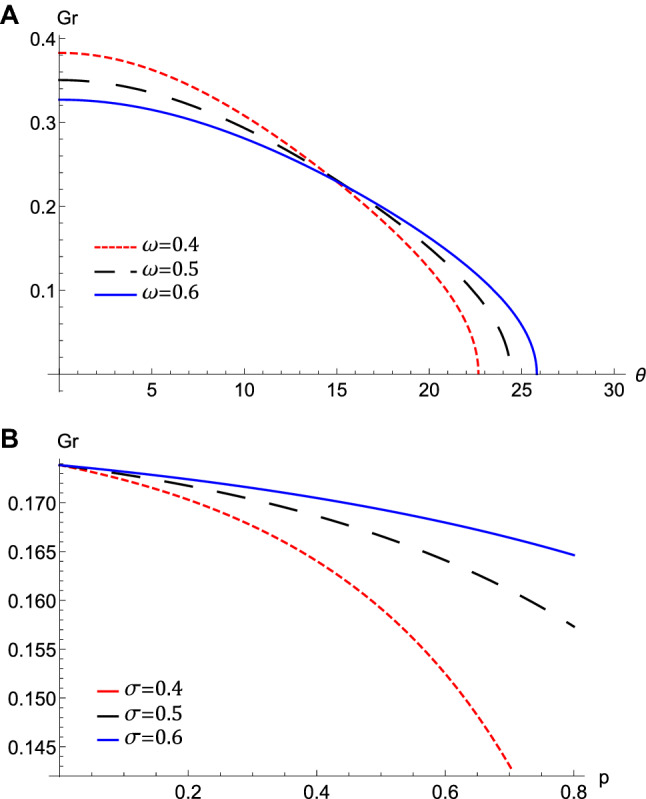
The variation of the growth rate, *Gr*, that represented by equation $\left( 30\right)$ at $l_{\xi }=0.7,l_{\eta }=0.4,M$ $=0.4$, $H_{e}=0.4$, and $H_{p}=0.6$, (**A**) against $\theta$ for different values of $\omega$ at $\sigma =0.2$, $p=0.4$, (**B**) against *p* for different values of $\sigma$ at $\omega =0.5$, $\theta =20$.

The nonlinear term *A* determines the amplitude and the polarity of the produced waves. The dependence of the nonlinear coefficient, *A* on $$\sigma $$ and *p* is illustrated in Fig. 2a. It is shown that, *A* is positive for all values of $$\sigma $$ and *p*. When both $$\sigma $$ and *p* change, we get different behavior. At first, *A* decreases as *p* increases till *p* reaches 0.1. Increasing the values of *p* over 0.1 leads to the increase of *A***.** Also, the behavior a gainst $$\sigma $$ takes two regions depending on *p* values. For $${\mathbf {0}}<p<0.5$$, it is found that *A* increases as $$\sigma $$ increases. The behavior is reversed for $$p>0.5$$, where *A* decreases as $$\sigma $$ increases. The effects of $$\sigma $$ and *p* on the properties of longitudinal, and the transverse dispersion coefficients, *B* and *C*, are manifested in Fig. 2b and c, respectively. Both coefficients decrease (increase) as *p* ($$\sigma $$) increases. The soliton solution Eq. (14) emerges due to the balance between the nonlinearity and the dispersion effects. The solitary wave amplitude, $$\phi _{m}$$ and width, *W*, are are also affected by the previous parameters, as shown in Fig. 3a and b, respectively. Since the amplitude depends on the nonlinear term, *A*, the amplitude is positive for all parameter values and it takes different behaviors depending on the *p* values, as depicted in Fig. 2a. At first, the amplitude increases as *p* increases from 0 to 0.1, at which point the amplitude reaches its maximum value. Increasing the values of *p* over 0.1 leads to a decrease in the amplitude. Furthermore, the amplitude varies with $$\sigma $$ differently against different *p* values. The amplitude decreases (increases) as $$\sigma $$ increases for $$p<0.5$$ ($$p>0.5$$). On the other hand, its width *W* is suppressed by increasing *p* or decreasing $$\sigma $$**.** The width *W* is also affected by the magnetic field through $$\omega $$ and rotation angle $$\theta $$ as depicted in Fig. 3c. The width increases (decreases) as $$\theta $$ ($$\omega $$) increases. The results obtained from Fig. 3 are confirmed by the solitary wave profiles and the associated electric field as shown in Figs. 4a–c and 5a–c, respectively. While the effects of electron and positron Bohm potentials on solitary waves are illustrated in Fig. 4d and e, respectively. It is shown that, since the electron and positron Bohm potentials, $$H_{e}$$ and $$H_{p} $$, respectively, appear only in both dispersion terms *B* and *C*, so they affect only the width of the QIASWs, while the amplitude remains constant for all values of both. The reason is that the amplitude depends only on the nonlinear term, which is not affected by $$H_{e}$$ or $$H_{p}$$ according to Eq. (11)**. **The increase of $$H_{e}$$ and $$H_{p}$$ leads to an increase in the width of the QIASWs. The associated electric fields are shown in Fig. 5d and e, respectively, and they show different behavior. The electric field amplitude depends on *W* which in turn depends on *B* and *C*. The wave energy depends mainly on the wave amplitude and width, as depicted in Eq. (19). Figure 6 shows that the energy is affected by the same parameters as they affect both $$\phi _{m}$$ and *W*. So the energy takes different behaviors against the change of $$\sigma $$ and *p* as was done in the case of $$\phi _{m}$$, and it is affected also by $$\omega $$ and $$\theta $$ in the same manner as both affect the width, *W*. Figure 6a shows that the energy increases (decreases) as *p* increases for $$p<0.2$$ ($$p>0.2$$ ). Also, the energy increases (decreases) as $$\sigma $$ increases for $$p<0.7$$ ($$p>0.7$$ ). The energy increases (decreases) as $$\theta $$ ($$\omega $$) as depicted in Fig. 6b. This is physically due to the stronger magnetic field leads to the confinement of the charged particles and consequently leads to a decrease in their energy. The variation of the inistability growth rate, *Gr*, against $$\sigma $$, *p*, $$\omega $$, and $$\theta 
$$ is illustrated in Fig. 7. It is obvious from Fig. 7a that *Gr* decreases from its maximum value to zero as $$\theta $$ increases. There is also a critical value of $$\theta $$ below (above) which *Gr* decreases (increrases) as $$\omega $$ increases. As $$\omega $$ decreases, the reduction of Gr against becomes more pronounced. Figure 7b shows that*Gr* increases (decreases) as $$\sigma $$(*p*) increases. We can see that in the high positron regime, the instability growth rate is an order of magnitude smaller than in the low positron concentration regime, as that found by Williams and Kourakis ^34^.

## Conclusions

The hydrodynamic model has been employed to investigate the QIASWs in a three-component collisionless quantum plasma consisting of electrons, positrons, and ions embedded in a uniform magnetic field. The ZK equation has been derived by employing the reductive perturbation method. The solution of this ZK equation has been used to explore the characteristics of the solitary wave. It is interesting that the soliton amplitude is positive for all values of the ions and positron densities and also for all electron and positron Fermi temperatures. The dependence of the soliton amplitude and width on the species densities and Fermi temperatures, the obliqueness, and the magnetic field (strength) were investigated. These effects have been verified in the case of soliton’s energy.

The instability analysis and investigation of the solitary wave solution have been performed. The plasma parameters’ effects on the instability growth rate have been discussed. It has been shown that an increase in positron Fermi temperature and density, magnetic field strength, and obliqueness can reduce the instability growth rate. Interestingly, we found that obliquity may cause soliton’s instability depletion as that resulted in the case of instability by Zedan et al.^33^. The obtained results of instability against system parameters are in agreement with those realized by El-Labany et al.^35^. An increase in the amount of electron trapping leads to a net shift towards higher values of the soliton energy. Particles trapped by a traveling plasma wave potential have received a significant amount of attention as a source of plasma wave instability. Sideband instability is an example of trapped particle instability ^36^. It has been discovered to be important in understanding the nonlinear saturation of deleterious stimulated light scattering processes in inertial confinement fusion^37,38^, as well as in astrophysical plasmas ^39^, where explanations for observed superthermal electrons and turbulent wave spectra are sought.

From the previous results, it is concluded that the energy is affected by the amplitude and width of the resultant waves. The energy has the same behavior as the amplitude when considering the effects of *p* and $\sigma$. While the energy adopts the width’s behavior when the effects of $\omega$ and $\theta$ are taken into account. According to Eq. (30), the instability growth rate, *Gr*, is affected by the wave amplitude and width in comparison with the energy in a different manner. At small $\theta$ values, $\theta <16^{\circ }$, both the energy and the instability growth rate decrease as $\omega$ increases. While for $\theta >16^{\circ }$, the energy still decreases, the instability growth rate acts oppositely where it increases as $\omega$ increases. Also $E_{{\mathbf {n}}}$ and *Gr* for $p<0.2$ both act differently against *p*, but both coincide in their behaviors for $p>0.2$ when large (small) values of positron (ion) concentration are attained. In this manner, considerable large energy is associated with a large instability growth rate. It is also shown that $E_{{\mathbf {n}}}$ (*Gr*) increases (decreases) as $\theta$ increases. For $p<0.7$, $E_{{\mathbf {n}}}$ (*Gr*) decreases (increases) as $\sigma$ increases. But for $p>0.7$, both increase as $\sigma$ increases. These behaviors against system parameters arise from the different equations that govern the instability growth rate and the energy.

Our findings are significant in understanding the physics of wave phenomena in e-p-i plasmas, which have recently received a lot of attention in astrophysical environments. Furthermore, the current study may be useful in explaining the phenomenon of particle acceleration in astrophysical environments because simulation studies show that the generation of electrostatic waves as a result of electrostatic instability plays a critical role in accelerating both electrons and positrons up to MeV ^40^. This may be possible if the kinetic energy of the plasma flow is effectively converted to electrostatic field energy, which can accelerate particles in a direction parallel to the background magnetic field ^41^.

Furthermore, the streaming instability of the Langmuir wave explains the pulsar magnetosphere radio emission^42^. The astrophysical magnetized plasmas can be treated as spin polarized. Thus, studying waves and instabilities while accounting for spin polarization may aid future research into the aforementioned manipulations. Although more simulation studies are needed to gain a better understanding of the obtained waves, our current work can be viewed as a first step toward the investigation of new wave energy and instabilities in laboratory plasmas such as semiconductor plasma^43^, high-intensity laser-solid matter interaction experiments^44^, in tokamaks ^45^ , and in space such as white dwarfs and pulsar magnetosphere ^46^ where e-p-i plasmas exist^6,47–54^ **.**

## Data Availability

The datasets used and analyzed during the current study are available from the corresponding author upon reasonable request.

## References

[1] Miller HR, Witta PJ (1987). Active Galactic Nuclei.

[2] Stenflo L, Shukla PK, Marklund M (2006). New low-frequency oscillations in quantum dusty plasmas. Europhys. Lett..

[3] El-Labany SK, El-Taibany WF, El-Samahy AE, Hafez AM, Atteya A (2016). Ion acoustic solitary waves in degenerate electron-ion plasmas. IEEE Transactions Plasma Sci..

[4] El-Monier SY, Atteya A (2019). Higher order corrections and temperature effects to ion acoustic shock waves in quantum degenerate electron-ion plasma. Chin. J. Phys..

[5] El-Taibany WF, Waidati M (2007). Nonlinear quantum dust acoustic waves in nonuniform complex quantum dusty plasma. Phys. Plasmas.

[6] Saha A, Pal N, Chatterjee P (2014). Dynamic behavior of ion acoustic waves in electron-positron-ion magnetoplasmas with superthermal electrons and positrons. Phys. Plasmas.

[7] El-Monier SY, Atteya A (2021). Dust-acoustic Gardner solitons in cryogenic plasma with the effect of polarization in the presence of a quantizing magnetic field. Zeitschrift für Naturforschung A.

[8] Lallement R, Welsh BY, Barstow MA, Casewell SL (2011). High ions towards white dwarfs: circumstellar line shifts and stellar temperature. Astron. Astrophys..

[9] Sabry R, Moslem WM, Shukla PK (2012). Freak waves in white dwarfs and magnetars. Phys. Plasmas.

[10] Ghosh N, Sahu B (2019). Nonlinear dispersive and dissipative electrostatic structures in two-dimensional electron-positron-ion quantum plasma. Commun. Theor. Phys..

[11] Haque Q (2020). Drift and ion acoustic waves in an inhomogeneous electron-positron-ion plasma with temperature degeneracy and exchange-correlation effects. Results Phys..

[12] Saha A, Pradhan B, Banerjee S (2020). Bifurcation analysis of quantum ion-acoustic kink, anti-kink and periodic waves of the Burgers equation in a dense quantum plasma. Eur. Phys. J. Plus.

[13] Iqbal Z, Khan IA, Khokhar TH, Murtaza G (2021). On the characteristics of magnetosonic waves in a spin-polarized degenerate electron-positron-ion plasma. IEEE Transactions Plasma Sci..

[14] Saha, A. & Banerjee, S.: Dynamical Systems and Nonlinear Waves in Plasmas., 1st Edition, Boca Raton, CRC Press (2021). 10.1201/9781003042549

[15] Samanta UK, Saha A, Chatterjee P (2013). Bifurcations of nonlinear ion acoustic travelling waves in the frame of a Zakharov–Kuznetsov equation in magnetized plasma with a kappa distributed electron. Phys. Plasmas.

[16] Hussain S, Imtiaz N, Hasnain H (2020). Oblique propagation of nonlinear solitary structures in electron positron ion plasmas under the influence of quantizing magnetic field. Plasma Res. Exp..

[17] Soltani H, Mohsenpour T, Sohbatzadeh F (2019). Obliquely propagating quantum solitary waves in quantum-magnetized plasma with ultra-relativistic degenerate electrons and positrons. Contributions Plasma Phys..

[18] Mohsenpour T, Ehsani H, Behzadipour M (2021). Ion-acoustic solitons in negative ion plasma with relativistic degenerate electrons and positrons. Waves Random Complex Media.

[19] Iqbal Z, Andreev PA, Murtaza GA (2019). A transverse separate-spin-evolution streaming instability and new wave solutions in electron-positron-ion plasmas. Astrophys. Space Sci..

[20] El-Shamy EF, Selim MM, El-Depsy A, Abdellahi MO, Al-Hagan O, Al-Mogeeth A, Alelyani L (2020). Effects of chemical potentials on isothermal ion-acoustic solitary waves and their three-dimensional instability in a magnetized ultra-relativistic degenerate multicomponent plasma. Phys. Plasmas.

[21] Khanum U, Iqbal Z, Murtaza G (2020). Hydrodynamic analysis of electrostatic counter-streaming instability in a spin-polarized electron-positron-ion plasma. Contributions Plasma Phys..

[22] Behery EE, Zaghloul MR (2021). Dynamics of electrostatic waves in relativistic electron-positron-ion degenerate plasma. Eur. Phys. J. Plus.

[23] Hongsit N, Allen MA, Rowlands G (2008). Growth rate of transverse instabilities of solitary pulse solutions to a family of modified Zakharov–Kuznetsov equations. Phys. Lett. A.

[24] Masood W, Mirza AM, Hanif M (2008). Ion acoustic shock waves in electron-positron-ion quantum plasma. Phys. Plasmas.

[25] El-Monier SY, Atteya A (2021). Propagation and energy of bright and dark solitons in magnetized quantum semiconductor plasmas in the presence of Bohm potential effect. Waves Random Complex Media.

[26] Allen MA, Rowlands G (1993). Determination of the growth rate for the linearized Zakharov–Kuznetsov equation. J. Plasma Phys..

[27] Allen MA, Rowlands G (1995). Stability of obliquely propagating plane solitons of the Zakharov–Kuznetsov equation. J. Plasma Phys..

[28] Mamun AA (1998). Instability of obliquely propagating electrostatic solitary waves in a magnetized nonthermal dusty plasma. Phys. Scr..

[29] Haider MM, Mamun AA (2012). Ion-acoustic solitary waves and their multi-dimensional instability in a magnetized degenerate plasma. Phys. Plasmas.

[30] Ko K, Kuehl HH (1978). Korteweg-de vries soliton in a slowly varying medium. Phys. Rev. Lett..

[31] El-Labany SK, El-Taibany WF, Behery EE (2013). Stability of three-dimensional dust acoustic waves in a dusty plasma with two opposite polarity dust species including dust size distribution. Phys. Rev. E.

[32] Fortov VE, Ivlev AV, Khrapak SA, Morfill GE (2005). Complex (dusty) plasmas: current status, open issues, perspectives. Phys. Rep..

[33] Zedan NA, Atteya A, El-Taibany WF, EL-Labany SK (2020). Stability of ion-acoustic solitons in a multi-ion degenerate plasma with the effects of trapping and polarization under the influence of quantizing magnetic field. Waves Random Complex Media.

[34] Williams G, Kourakis I (2013). On the existence and stability of electrostatic structures in non-Maxwellian electron-positron-ion plasmas. Phys. Plasmas.

[35] El-Labany SK, Moslem WM, Elneely NK (2020). Stability of obliquely propagating 3D solitons in magnetized plasma with nonthermal distribution. Adv. Space Res..

[36] Kruer WL, Dawson JM, Sudan RN (1969). Trapped-Particle Instability. Phys. Rev. Lett..

[37] Brunner S, Valeo EJ (2004). Trapped-particle Instability Leading to Bursting in Stimulated Raman Scattering Simulations. Phys. Rev. Lett..

[38] Krasovsky VL (2009). Classification of sideband instability regimes for whistler waves with trapped electrons. Plasma Phys. Controlled Fusion.

[39] Dodin IY, Schmit PF, Rocks J, Fisch NJ (2013). Negative-mass instability in nonlinear plasma waves. Phys. Rev. Lett..

[40] Khanum U, Iqbal Z, Murtaza G (2020). Hydrodynamic analysis of electrostatic counter-streaming instability in a spin-polarized electron-positron-ion plasma. Contrib. Plasma Phys..

[41] Saito S, Sakai JI (2004). Particle acceleration during the counterstreaming instability in magnetized pair plasmas. Phys. Plasmas.

[42] Asseo E, Melikidze GI (1998). Non-stationary pair plasma in a pulsar magnetosphere and the two-stream instability. Mon. Not. R. Astron. Soc..

[43] Shukla PK, Rao NN, Yu MY, Tsintsadze NL (1986). Relativistic nonlinear effects in plasmas. Phys. Rep..

[44] Berezhiani V, Tskhakaya DD, Shukla PK (1992). Pair production in a strong wake field driven by an intense short laser pulse. Phys. Rev. A.

[45] Helander P, Ward DJ (2003). Positron creation and annihilation in tokamak plasmas with runaway electrons. Phys. Rev. Lett..

[46] Verdon MW, Melrose DB (2008). Wave dispersion in a counterstreaming, cold, magnetized, electron-positron plasma. Phys. Rev. E.

[47] Irfan M, Ali S, Mirza AM (2014). Dust-acoustic solitary and rogue waves in a Thomas-Fermi degenerate dusty plasma. Astrophys. Space Sci..

[48] Irfan M, Ali S, Ata-ur-Rahman, Mirza AM (2019). Arbitrary amplitude oblique electrostatic solitary waves in a degenerate cold dusty magnetoplasma. IEEE Transactions Plasma Sci..

[49] Abd-Elzaher M, Atteya A (2021). Obliquely overtaking collisions of electrostatic N-soliton in the Thomas-Fermi dense magnetoplasma. Waves Random Complex Media.

[50] Jehan N, Salahuddinin M, Mahmood S, Mirza AM (2009). Electrostatic solitary ion waves in dense electron-positron-ion magnetoplasma. Phys. Plasmas.

[51] Chatterjee P, Saha T, Muniandy SV, Yap SL, Wong CS (2009). Solitary waves and double layers in dense magnetoplasma. Phys. Plasmas.

[52] Rahim Z, Adnan M, Qamar A, Saha A (2018). Nonplanar dust-acoustic waves and chaotic motions in Thomas Fermi dusty plasmas. Phys. Plasmas.

[53] Roy D, Ghosh N, Sahu B (2020). Nonlinear modulation of quantum electron acoustic waves in a Thomas-Fermi plasma with effects of exchange-correlation. Indian J. Phys..

[54] Atteya A, El-Borie MA, Roston GD, El-Helbawy AS, Prasad PK, Saha A (2021). Ion-acoustic stable oscillations, solitary, periodic and shock waves in a quantum magnetized electron-positron-ion plasma. Zeitschrift für Naturforschung A.

